# Parylene-C Modified OSTE Molds for PDMS Microfluidic Chip Fabrication and Applications in Plasma Separation and Polymorphic Crystallization

**DOI:** 10.3390/bios15060388

**Published:** 2025-06-16

**Authors:** Muyang Zhang, Haonan Li, Xionghui Li, Zitong Ye, Qinghao He, Jie Zhou, Jiahua Zhong, Hao Chen, Xinyi Chen, Yixi Shi, Huiru Zhang, Lok Ting Chu, Weijin Guo

**Affiliations:** 1 MOE Key Laboratory of Tumor Molecular Biology, Jinan University, Guangzhou 510632, China; 2 Department of Biomedical Engineering, Shantou University, Shantou 515063, China; 3 Guangdong Foshan Lianchuang Graduate School of Engineering, Foshan 528311, China; 4 Institute of Biochemistry and Molecular Biology, Guangdong Medical University, Zhanjiang 524023, China

**Keywords:** OSTE, parylene-C, microfluidic chip, plasma separation, polymorphic crystallization

## Abstract

This work presents a novel microfabrication process that addresses the interference of thiol groups on off-stoichiometry thiolene (OSTE) surfaces with the curing of polydimethylsiloxane (PDMS) by integrating the high-performance polymer Parylene-C. The process utilizes a Parylene-C coating to encapsulate the active thiol groups on the OSTE surface, enabling precise replication of PDMS microstructures. Based on this method, PDMS micropillar arrays and microwell arrays were successfully fabricated and applied in passive plasma separation and polymorphic crystal formation, respectively. The experimental results demonstrate that the plasma-separation chip efficiently isolates plasma from whole-blood samples with varying hematocrit (HCT) levels, achieving a separation efficiency of up to 57.5%. Additionally, the microwell array chip exhibits excellent stability and controllability in the growth of salt and protein crystals. This study not only provides a new approach for microfabricating microfluidic chips, but also highlights its potential applications in biomedical diagnostics and materials science.

## 1. Introduction

Microfluidics technologies have emerged as transformative tools in modern scientific research and industrial applications, aiming to enable precise manipulation and analysis of fluids and materials at the microscale and nanoscale. Microfluidics has revolutionized fields such as biomedical diagnostics, chemical synthesis, and environmental monitoring [1]. With continuous advancements in microfluidics, multiple laboratory functions can now be integrated onto a single chip, often referred to as a “lab-on-a-chip”. This integration significantly reduces sample and reagent consumption, enhances analytical speed, and improves experimental reproducibility [2]. Complementing microfluidics, microfabrication techniques provide foundational methods for creating complex microstructures with high precision and scalability. These techniques, including photolithography, soft lithography, and etching processes, have enabled the development of advanced microfluidic devices, which play critical roles in applications like biosensing, tissue engineering, and drug delivery [3]. The synergy between microfluidics and microfabrication has further expanded the possibilities for designing multifunctional platforms to address complex challenges in healthcare, energy, and materials science [4]. The emergence of materials used in microfabrication plays a crucial role in diversifying the functionality, performance, and fabrication processes of microfluidic devices [5].

Silicon is widely used in microfluidic chip fabrication due to its excellent mechanical stability, high precision, and compatibility with semiconductor manufacturing processes [6]. Glass is favored for its thermal stability, chemical resistance, and optical transparency, making it ideal for applications like microscopy, electrophoresis, and cell culture [7]. However, the microfabrication of silicon and glass needs cleanroom facilities, and the cost is high. Thermosetting plastics, such as SU-8 photoresist, epoxy, and PDMS, are widely used due to their excellent moldability, chemical resistance, and biocompatibility, making them ideal for rapid prototyping and organ-on-a-chip applications [8]. SU-8, a negative photoresist, has been extensively used for creating molds for soft lithography, taking advantage of its excellent mechanical durability and microfabrication properties [9]. Xia and Whitesides pioneered the use of PDMS in soft lithography, enabling the rapid prototyping of microfluidic devices with high precision [3]. In comparison, thermoplastics like PMMA, PC, PS, and COC are also widely used because of their low cost, ease of mass production (e.g., by injection molding or hot embossing), and good optical properties, making them suitable for point-of-care diagnostics and high-throughput screening [10]. Nevertheless, generally, the surface modification of thermoplastics is complicated with a multi-step process, which hinders their further applications [11,12]. With the fast development of 3D printing technology, 3D printing resins (e.g., acrylics, PDMS-like resins, and biocompatible photopolymers) enable rapid, customizable microfluidic chip fabrication with complex geometries, making them ideal for rapid prototyping, organ-on-a-chip models, and bespoke lab-on-a-chip systems [13]. But some resins have poor biocompatibility and require post-processing to reduce cytotoxicity [14,15]. In addition, the poor gas permeability of resins makes them unsuitable for cell culture or long-term biological applications [16,17]. Gelatin, with superior biocompatibility and tunable mechanical properties, has also been used for the fabrication of microfluidic chips with cell-laden applications [18,19]. However, its poor mechanical stability, low fabrication resolution, and difficult integration with other materials limit its applications in other biomedical fields [20].

Replica molding is a cornerstone technique in microfluidic chip fabrication, offering high precision, cost-effectiveness, and scalability for creating complex microstructures like channels, pillars, and wells [21,22]. Widely used for the rapid prototyping of PDMS-based lab-on-a-chip and organ-on-a-chip devices, this method enables the replication of sub-micron features from master molds while supporting multi-material integration with elastomers, hydrogels, and thermoplastics [23,24,25]. Its advantages include its low cost (minimizing cleanroom steps), high fidelity for intricate patterns, and flexibility for both disposable and reusable diagnostic devices. While replica molding has been advanced using SU-8, thermoplastics, and hydrogels, these materials face trade-offs between resolution, reusability, and chemical compatibility [26]. SU-8 requires silanization for PDMS release, thermoplastics lack elastomeric properties, and hydrogels suffer from poor mechanical stability. Therefore, other material alternatives are needed to improve the overall performance of replica molding.

Recently, off-stoichiometry thiolene (OSTE) has been introduced as a novel polymer for the fabrication of microfluidic devices [27]. OSTE is a polymer in which thiol monomers and alkene monomers are mixed through stoichiometrically non-conforming ratios and cured by click chemistry under UV light irradiation [27]. OSTE is characterized by a hard and tough nature, which renders it suitable for utilization as a microfluidic chip material or a mold for inverting other materials, including gelatine and agarose gels [28,29]. In comparison with the prevalent microfluidic material PDMS, OSTE demonstrates superior precision and efficiency in the fabrication of microfluidic chips, and its excellent chemical and mechanical properties have long been demonstrated in many studies [30,31,32,33]. However, as a microfluidic mold, the thiol groups on the surface of OSTE make it difficult for replica molding of PDMS. The thiol groups will be the first to inactivate the curing agent by a strong ligand interaction when covering PDMS prepolymer on OSTE, which will inhibit the silica–hydrogen addition reaction during the curing of PDMS. To solve this problem and extend the applications of OSTE in the fabrication of microfluidic devices, Parylene-C was integrated with the microfabrication process of OSTE to achieve the encapsulation of thiol groups on the OSTE surface in this work.

Parylene-C, a high-performance polymer coating material, exhibits exceptional physicochemical properties, including high electrical insulation, corrosion resistance, biocompatibility, and extremely low permeability [34]. Compared with other Parylene derivatives, Parylene C demonstrates superior moisture resistance and chemical stability due to the presence of chlorine substituents [35]. Parylene-C is deposited through a solvent-free chemical vapor deposition process, where monomers polymerize on substrates to form conformal, pinhole-free coatings with controllable thickness (nanometers to micrometers). Parylene-C has demonstrated irreplaceable advantages in applications such as microelectronic packaging [36], biomedical device protection [37,38], aerospace and marine corrosion prevention [39], and even cultural heritage preservation [40], due to its unmatched conformality and biocompatibility.

By integrating a Parylene-C coating, we developed a novel microfabrication process by replica molding of OSTE molds. The method involves encapsulating active thiol groups on OSTE via chemical vapor deposition of Parylene-C, enabling high-fidelity replica molding of PDMS microstructures. While OSTE and Parylene-C have been individually explored in prior research, their integration to overcome the interference of thiol groups on OSTE surfaces during PDMS curing has not been reported previously. Using this approach, we successfully fabricated PDMS micropillar and microwell arrays, which were applied to passive plasma separation devices and polymorphic crystal growth platforms as proof-of-concept, respectively. Plasma separation is critical for point-of-care diagnostics as it enables the rapid isolation of cell-free biomarkers (e.g., proteins and nucleic acids) from whole blood, eliminating interference from cellular components and ensuring assay accuracy [41]. The microwell array platform is used to generate NaCl and glycine crystals, which have significant real-world applications across multiple industries. NaCl crystals are essential for saline-based injectables and oral rehydration solutions in pharmaceuticals, and serve as flavor enhancers and preservatives in the food industry [42,43]. Glycine crystals, on the other hand, play a pivotal role in pharmaceutical polymorphism studies, where different polymorphs affect drug stability, solubility, and efficacy—particularly critical for optimizing formulations [44,45]. This work not only provides a robust solution for PDMS replication from OSTE molds but also highlights the practical potential of the developed devices in point-of-care diagnostics (e.g., blood plasma isolation) and materials science (e.g., high-throughput polymorphic crystallization). The integration of Parylene-C’s chemical stability with OSTE’s mechanical precision offers a reusable, scalable fabrication platform, bridging a critical gap in microfluidic device manufacturing.

## 2. Materials

Polydimethylsiloxane (PDMS, Sylgard 184) was obtained from Dow Corning (Midland, MI, USA). The blood filtration membrane was obtained from Cobetter (Hangzhou, China). The hydrophilic tape was provided by Adhesives Research (Shanghai, China). The hydrophobic correction fluid was obtained from Pentel (Tokyo, Japan). The whole blood samples were obtained from Junma Biotechnology (Taiyuan, Shanxi, China). Sodium chloride (NaCl) and glycine were obtained from Macklin Biochemical (Shanghai, China). The NaCl solution for crystallization was 36% (saturated), 27%, 18%, and 9% NaCl in DI water. The glycine solution was 25% glycine in DI water for 20 °C crystallization, 29% glycine in DI water for 25 °C crystallization, 37% glycine in DI water for 35 °C crystallization, and 54% glycine in DI water for 50 °C crystallization. The mineral oil was from Shengyang Chemical (Weihui, Henan, China).

The OSTE prepolymer (OSTE40) was configured and prepared by extracting the bubbles with a vacuum pump in a light-avoiding environment [27]. A 7.5 cm × 2.5 cm glass slide was selected as the carrier for the OSTE structure and was plasma-hydrophilized with an oxygen plasma cleaner before the experiment. Three kinds of plastic film of different dimensions were used in the experiment. Plastic film A was 3.5 cm × 8.5 cm with a thickness of 100 µm, and treated with a hydrophobic spray. Plastic film B was 2.5 cm × 0.7 cm with a thickness of 100 µm. Plastic sheet C was 2.5 cm × 0.7 cm with a thickness of 350 µm. Propylene glycol methyl ether acetate (PGMEA) was selected as the developer for OSTE, and isopropanol was utilized as the cleaner after PGMEA development.

## 3. Experiments

### 3.1. Microfabrication of OSTE

**Figure 1:**
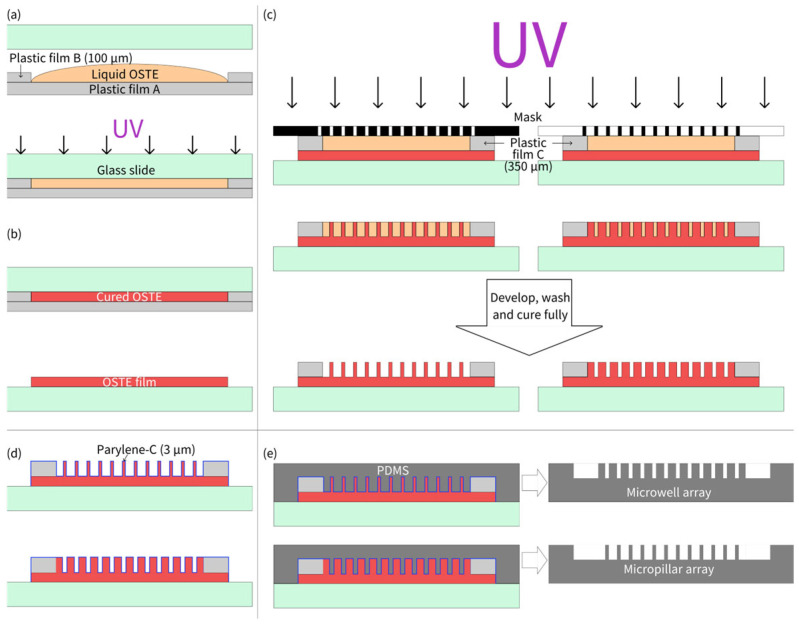
(**a**) Thickness definition of OSTE film using plastic film supports. (**b**) Plastic film peeling off after UV curing. (**c**) Lithography of OSTE prepolymer with mask-defined microstructures. (**d**) Parylene-C coating on OSTE microstructures. (**e**) PDMS microstructure replication by OSTE molds coated with Parylene-C.

The fabrication process of the OSTE micropillar and microwell array is shown as Figure 1. As illustrated in Figure 1a, two pieces of plastic film B were first placed on the hydrophobic side of plastic film A to serve as supports. The OSTE prepolymer was then dropped between two pieces of plastic film B. A hydrophilic glass slide was pressed onto plastic film B, and after the OSTE prepolymer diffused to fill the gap, the entire setup was placed in a photolithography equipment for a 10 s exposure with a UV light intensity of 7.65 mW/cm^2^. After removing plastic film A, due to its hydrophobic surface and the hydrophilic surface of the glass slide, the cured OSTE adhered to the glass slide, forming an OSTE film with the same thickness as that of plastic film B (100 μm). Subsequently, plastic film C was mounted at both ends of the OSTE film on the glass slide, and OSTE prepolymer was poured onto the substrate again. A photomask was then pressed face-down onto plastic piece C, and after the OSTE prepolymer diffused to fill the gap, the entire setup was exposed for 7 s for micropillar array fabrication (3 s for microwell array fabrication). Different UV exposure times were used for micropillar and microwell arrays with the same thickness. For micropillars, we usually overexposed the microstructures to ensure the strength of pillars during development (to avoid collapse), while overexposure was not needed for microwells. After exposure, the photomask was separated from the formed structure. The structure was developed using PGMEA, with the development time being ~2 min. Residual PGMEA was then rinsed off with isopropanol. The cleaned structure was left to air-dry and then post-cured under UV light for 3 min, completing the fabrication of the OSTE micropillar or microwell array. After that, the prepared OSTE structure was then coated with Parylene-C by chemical vapor deposition (VZ-PC1, Dongguan Weijing Nanomaterials, Dongguan, China), with a thickness of 3.0 μm, measured and verified by Dongguan Weijing Nanomaterials. Parylene-C coating of 3.0 μm can ensure that the thiol groups do not have any influence on the curing of PDMS, and also do not lose the fidelity of microstructures during replica molding. Then, PDMS microstructures can be fabricated through replica molding of OSTE coated with Parylene-C following the standard soft lithography protocol [3].

### 3.2. Micropillar Array for Plasma Separation

The PDMS micropillar array fabricated using the aforementioned process was cut into a T-shaped pattern (consisting of a 1.0 cm × 1.0 cm square and a 2.0 cm × 0.3 cm rectangle) and subjected to plasma treatment to enhance its hydrophilicity. The dimension of micropillars was 200 μm in diameter, 350 μm in height, and 500 μm in center-to-center distance. The blood filtration membrane was a specialized filter membrane with a unique asymmetric pore structure, featuring different pore sizes on its two sides. This design effectively blocked blood cells on the side with larger pores while allowing plasma to filter through the side with smaller pores, making it a commonly used membrane for plasma separation. However, during use, blood cells from whole blood samples tended to flow out from the sides of the membrane, mixing with the separated plasma. To address this issue, a hydrophobic barrier was created on the edges of the membrane using correction fluid to prevent lateral flow of the sample. A 1.0 cm × 1.0 cm square blood filtration membrane was cut, and one side of the membrane was vertically immersed into the correction fluid on the surface. After the correction fluid was fully absorbed and dried, the same procedure was repeated for the remaining sides of the membrane, resulting in a blood filtration membrane with hydrophobic barriers on all four edges. A hydrophilic adhesive tape (2.5 cm × 0.3 cm) was prepared for covering the rectangle channel.

### 3.3. Microwell Array for Formation of Salt and Protein Crystals

For the formation of salt crystals and protein crystals, microwell arrays prepared by the process described above were used. Here, we used salt crystallization for the illustration of the experimental procedures. The NaCl solution was prepared at 25 °C and the PDMS microwell array was placed in a vacuum chamber to degas it for 30 min. Because of the inherent properties (mainly gas permeability) of PDMS, after vacuum treatment, the PDMS could absorb air to create negative pressure [46]. Immediately after the degas process, the NaCl solution is poured onto the microwell array and an air cavity was formed between water and PDMS. As the PDMS absorbed air, within 5 min, the solution on its surface was sucked into the microwell under negative air pressure. After that, residual solution on the PDMS surface was removed gently using a pipette, ensuring minimal disturbance to the confined droplets. Then, the surface of the microwell array was immediately sealed with mineral oil. The oil was poured slowly at the edge of the chip and allowed to spread uniformly, avoiding turbulence. Under the influence of surface tension of the oil–water interface, isolated droplets were formed inside the microwells. The oil’s low density ensured that it flowed over the PDMS surface without displacing the denser aqueous droplets, which remained anchored due to the PDMS’s hydrophilic plasma-treated walls and the capillary force ensured by the microwells’ aspect ratio. Due to its immiscibility with aqueous solutions and low interfacial tension, the oil layer acts as a protective barrier, preventing displacement during cleaning while enabling evaporation control for crystallization. The method’s efficacy is demonstrated by the high success rate of crystal formation, with minimal cross-contamination or droplet loss observed.

## 4. Results

### 4.1. OSTE Micropillar and Microwell Array

**Figure 2:**
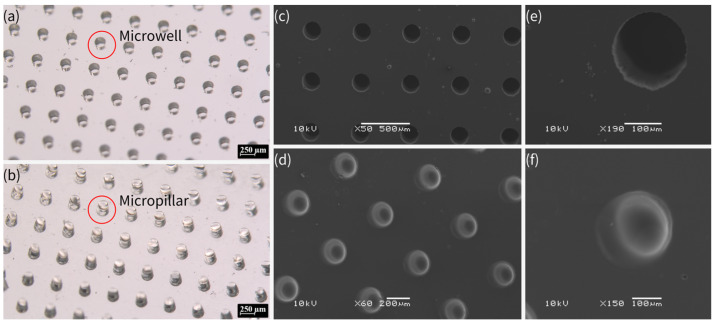
(**a**) PDMS microwell array in orthogonal arrangement under stereomicroscope, with dimensions of 200 μm in diameter and 350 μm in depth, and center-to-center pitch distance of 500 μm. (**b**) PDMS micropillar array in orthogonal arrangement under stereomicroscope, with dimensions of 200 μm in diameter and 350 μm in height, and center-to-center pitch distance of 500 μm. (**c**–**f**) SEM images of PDMS microwell array (**c**,**e**: with dimensions of 200 μm in diameter and 350 μm in depth) and micropillar array (**d**,**f**) with dimensions of 200 μm in diameter and 350 μm in height). The scale bars indicate 250 μm in (**a**,**b**), 500 μm in (**c**), 200 μm in (**d**), and 100 μm in (**e**,**f**).

Figure 2 shows pictures of the PDMS micropillar and microwell array fabricated by this process under stereomicroscopy and scanning electron microscopy (SEM). The fabrication process of PDMS micropillar and microwell arrays involved a precise, multi-step lithographic approach, which is similar to PDMS replica molding via soft lithography using an SU-8 mold by standard lithography. However, in this work, OSTE after lithography was treated by Parylene-C, whereas SU-8 was treated by silanization to facilitate the peeling off of PDMS. This method ensured high-fidelity replication of microstructures, as confirmed by stereomicroscopy and SEM imaging. The Parylene-C modification was critical to prevent PDMS curing inhibition by OSTE’s thiol groups, facilitating the production of functional PDMS chips for following plasma separation and crystallization applications. The process combines UV lithography’s precision with material innovation, offering a robust platform for microfabrication. In addition, due to the superior adhesion and stability of Parylene-C coating, the OSTE mold coated with Parylene-C could be reused for replica molding with consistent fidelity. According to testing in our lab, OSTE molds coated with Parylene-C could be reused at least 20 times without structural or functional damage.

In comparison with the commonly used SU-8 master mold, OSTE’s UV-curability allows for sub-micro features [30], rivaling SU-8, but with reusability. Parylene-C deposition is solvent-free and conformal, suitable for industrial-scale microfabrication, with superior durability, allowing for multiple replication cycles without feature degradation or damage [47,48]. However, SU-8 would experience damage or loss of small structures (such as sharp corners) due to its inherent brittle properties after multiple replications [49,50], while OSTE is a highly crosslinked polymer with a stronger toughness and can resist more replications [51,52]. In addition, this approach in our work is compatible with other materials, including hydrogels and other polymers, broadening its application scope.

The lowest precision (smallest reliable feature size) of microstructures fabricated using the technique in this work was approximately 100 µm, but we believe the precision of this approach has the potential to reach sub-10 µm or smaller under optimized conditions. It has been already shown that OSTE can be used to fabricate sub-micro microstructures [30]; therefore, by combination with robust Parylene-C coating, it should be easy to achieve sub-10 µm precision using this approach. Future research could explore the optimization of Parylene-C coating thickness to further enhance the fidelity of PDMS replication, particularly for sub-10 µm microstructures. Additionally, extending this fabrication method to other elastomers or biocompatible materials beyond PDMS could broaden its applicability in organ-on-a-chip and implantable devices. Investigating the long-term stability of Parylene-C-coated OSTE molds under repeated use or harsh chemical conditions would also be valuable for industrial-scale adoption. Finally, integrating this technique with advanced lithography methods, such as two-photon polymerization, could enable the fabrication of hierarchical microstructures for more complex microfluidic applications.

### 4.2. Passive Plasma Separation

**Figure 3:**
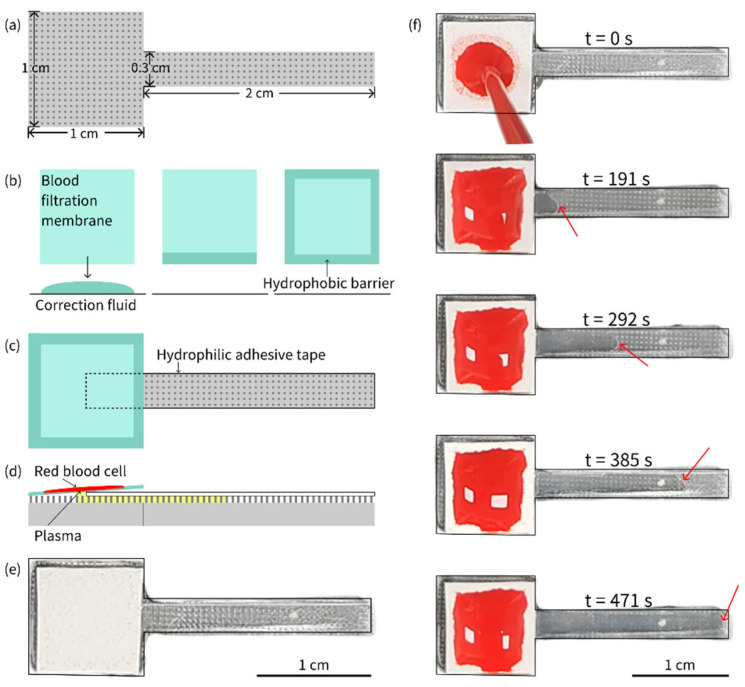
(**a**) Shape and size of the PDMS piece with a micropillar array. (**b**) Preparation of hydrophobic barriers on the blood filtration membrane. (**c**) Plasma separation device assembled from a PDMS piece with a micropillar array, a filtration membrane and a hydrophilic adhesive tape. (**d**) Schematic of plasma separation from whole blood sample on the device. (**e**) Real image of the plasma separation device. (**f**) Plasma separation from an 80 µL whole blood sample with 55% HCT on the device. The separated plasma was pumped continuously until it completely filled the gap between the hydrophilic tape and the micropillar array. The red arrow indicates the position of the plasma front.

A PDMS micropillar array (as shown in Figure 3a), a blood filtration membrane with hydrophobic barriers (as shown in Figure 3b), and a piece of hydrophilic adhesive tape were assembled into a plasma separation device, as depicted in Figure 3c,d, which shows a schematic of the cross-section of the plasma separation device, whereas Figure 3e shows a real image of the plasma separation device. A series of images of plasma separation from whole blood is presented in Figure 3f.

Whole blood samples with hematocrit (HCT) levels of 30%, 35%, 40%, 45%, 50%, 55%, and 60% were prepared by adding or extracting plasma after centrifugation. These samples were then used for plasma separation using the fabricated device. Each experiment was repeated at least three times. Through parameter optimization by multiple experiments, it was demonstrated that, when the volume of whole blood samples was set at 80 μL, the plasma separated from whole blood samples with varying HCT levels could fully fill the device, achieving the goal of plasma collection with consistent volume. When the plasma completely filled the micropillar array, the gaps within the micropillar array could be used to refer to the actual collected plasma (only the rectangular part of the T-shaped device was considered), allowing for the estimation of the plasma separation efficiency:

$$E_{p}=\frac{V_{G}}{V_{S}\times \left(1-HCT\right)}$$

where $V_{G}$ is the volume of the gap in the micropillar array and $V_{S}$ is the volume of whole blood sample. The gap volume can be estimated:

$$V_{G}=H\times \left(S_{R}-S_{C}\times N\right)$$

where H is the height of the micropillar array, $S_{R}$ is the total area of the rectangle, $S_{C}$ is the sum of the base areas of the micropillar array, and N is the total number of micropillars in the rectangular part. The number of micropillars can be calculated using the following formula:

$$N=\frac{L}{D_{C}+D_{S}}\times \frac{W}{D_{C}+D_{S}}$$

where L is the length of the rectangle, W is the width of the rectangle, $D_{C}$ is the diameter of the micropillar, and $D_{S}$ is the spacing between micropillars. The equations mentioned above were deduced by the authors ourselves. Based on the above formulas, the gap volume in a rectangular region of 0.3 cm × 2.0 cm was approximately 18.4 µL. For a whole blood sample, even with a high HCT of 60%, the plasma separation efficiency could reach 57.5%, which was similar to or higher than that in previous reports [53,54].

The microfluidic device developed in this study demonstrated excellent performance in passive plasma separation, achieving high efficiency and consistent performance. The dimensions of the micropillar and micropillar array layouts were mainly designed based on our previous work [55]. The device consistently separated plasma across a wide range of HCT levels (30–60%) using an optimized sample volume of 80 µL, ensuring uniform plasma collection. Key innovations included a hydrophobic barrier applied to the edges of the blood filtration membrane to prevent cell contamination and a precisely fabricated PDMS micropillar array that enabled efficient, power-free separation. The separation efficiency was quantitatively validated by calculating the gap volume within the micropillar array, confirming the reliability of the design. In comparison, this microfluidic device for plasma separation combined the broad HCT adaptability of cotton threads [53], the high efficiency of crossflow designs [54], the simple design of an OSTE pillar forest [55], and the rapid processing of synthetic paper [56], while leveraging a scalable fabrication process. Its integrated hydrophobic barriers and potential for multiplexing further distinguished it as a robust solution for passive plasma separation. With its simplicity, high performance, and passive operation, this microfluidic platform holds great potential for point-of-care diagnostics, particularly in resource-limited settings, showcasing the successful integration of advanced materials (Parylene-C, OSTE, and PDMS) and innovative engineering to address challenges in biomedical sample processing. In addition, by removing the filtration membrane and centrifugating the other parts of the device using a customized setup [56], all of the plasma separated can be collected into a tube for subsequent storage and analysis if needed. Future optimization will focus on quantifying protein recovery and hemolysis to fully benchmark against state-of-the-art methods.

### 4.3. Crystallization of NaCl

**Figure 4:**
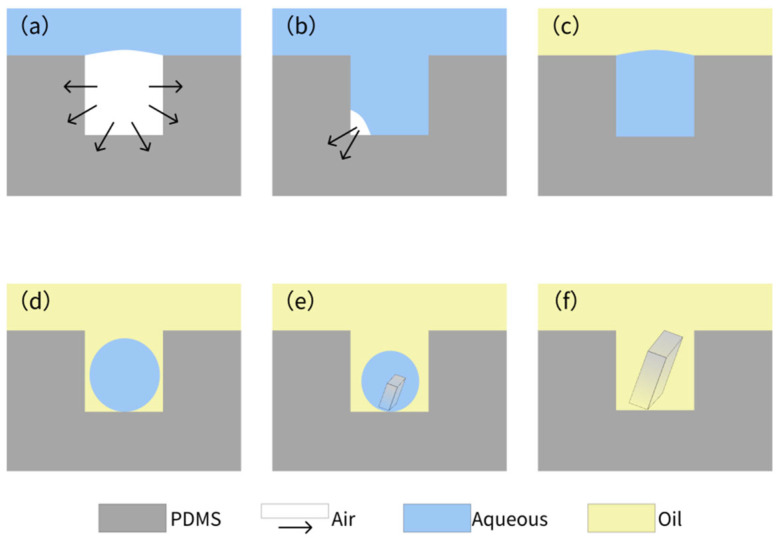
Air absorption by the degassed PDMS (**a**) and gradual filling of solution under negative pressure (**b**). (**c**) Encapsulation of the solution in the microwell array, covered by the mineral oil. (**d**) Formation of droplets in the microwell array due to surface tension. (**e**,**f**) Formation of crystals within the microwell.

**Figure 5:**
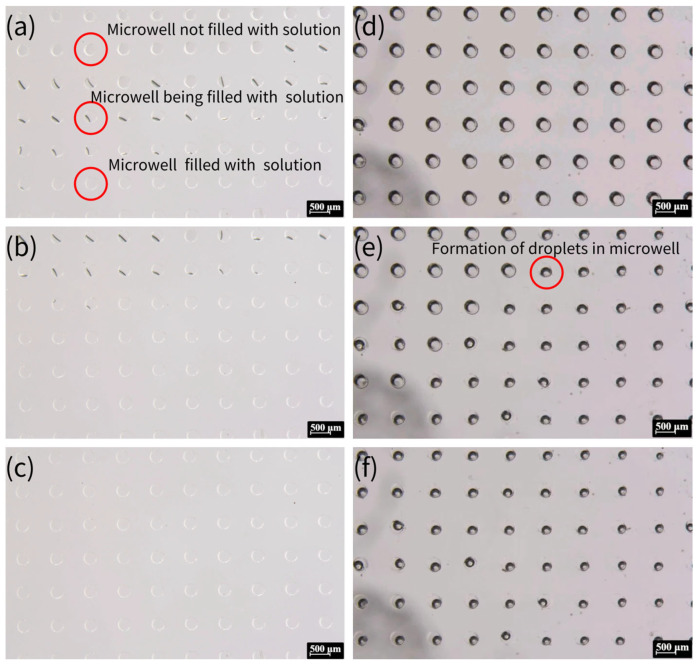
The process of solution being drawn into the microwells and the formation of oil-encapsulated droplets. (**a**) Aqueous solution being drawn into the microwells via negative pressure generated by degassed PDMS. The top section displays empty microwells, the middle section shows the liquid front advancing (indicated by the black line), and the bottom section depicts fully filled microwells. (**b**) After approximately 30 s, additional microwells are filled, and the liquid surface becomes clearly defined. (**c**) All microwells filled with aqueous solution within 3 min. (**d**) The microwell array surface covered by mineral oil. (**e**) Droplet formation within some microwells due to interfacial tension after 5 min. (**f**) Final state where all microwells contain isolated droplets, ready for crystallization.

The whole process of crystallization is shown in Figure 4, which shows a typical process for NaCl crystallization. This protocol ensures reproducible droplet isolation, critical for high-throughput crystallization. Figure 5 illustrates the critical process of solution confinement within the microwell array, which is essential for controlled crystal formation. The shape and dimensions of the microwells have a significant influence on the crystal formation. The microwell dimensions directly affect the confinement and evaporation dynamics of the solution, which are critical for controlled nucleation and crystal growth. The height and diameter of the microwells influence the droplet volume and interfacial tension, which in turn affect the nucleation sites and crystal morphology. The uniformity of the microwells enables reproducible droplet isolation, minimizing variability in crystal formation. The dimensions of the microwell and microwell array layouts were mainly designed based on a previous report [57]. The experiment demonstrated how degassed PDMS generated negative pressure to draw the aqueous solution into the microwells (Figure 5a–c), followed by mineral oil encapsulation to stabilize the isolated droplets via interfacial tension (Figure 5d–f). This experiment was vital for validating the device’s ability to achieve reproducible droplet isolation, a cornerstone of controlled crystallization. Without proper confinement, solutions would evaporate unevenly or mix, leading to inconsistent nucleation and crystal growth. The negative pressure-driven filling and oil encapsulation ensured that each microwell functioned as an independent microreactor, enabling parallelized and standardized conditions for crystal formation.

**Figure 6:**
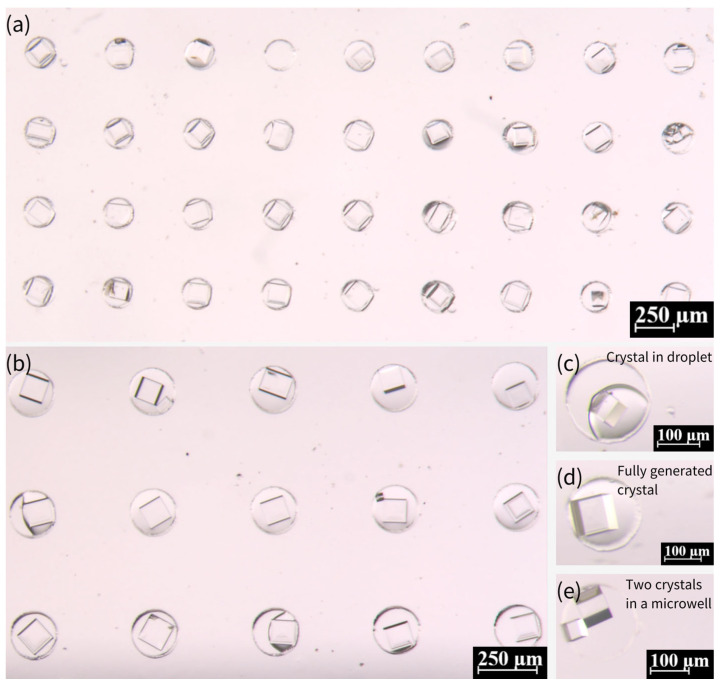
Morphology and growth of NaCl crystals in PDMS microwell arrays. (**a**,**b**) NaCl crystals formed within microwells using a saturated solution (0.36 g/mL), exhibiting well-defined cubic structures. (**c**) Transitional state showing coexistence of droplets and small crystals in some microwells during growth. (**d**) Fully developed NaCl crystals with clear cubic morphology after one week. (**e**) Rare instance of dual small crystals nucleating in a single microwell. Scale bars: 250 μm in (**a**,**b**); 100 μm in (**c**–**e**).

Figure 6 presents the generation of crystals from 0.36 g/mL NaCl solution within microwells. Most of the microwells show the existence of a single crystal, while two crystals existed in very few microwells. The presence of single versus multiple crystals in microwells can be attributed to nucleation dynamics, droplet confinement, surface interactions, and experimental conditions. Single crystals typically form when nucleation is controlled and limited to a single site, often under moderate supersaturation and uniform evaporation rates. In contrast, multiple crystals arise when higher supersaturation, localized concentration variations, or rough surfaces promote nucleation at multiple sites. Droplet stability also plays a key role—well-confined droplets with homogeneous surfaces favor single crystals, while uneven evaporation or heterogeneous interfaces may lead to multiple nucleation events. Additionally, stochastic factors, such as minor variations in droplet volume or impurities, contribute to this variability. Thus, while optimizing conditions can improve uniformity, microscale crystallization inherently exhibits some variability due to the complex interplay of these factors.

**Figure 7:**
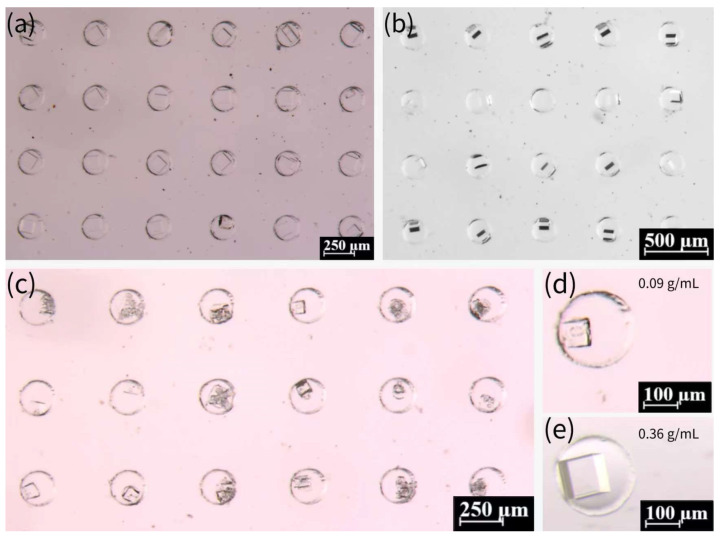
(**a**) Crystals prepared from 0.27 g/mL NaCl solution in microwells. (**b**) Crystals prepared from 0.18 g/mL NaCl solution in microwells. (**c**) Crystals prepared from 0.09 g/mL NaCl solution in microwells. (**d**) Crystals prepared from 0.09 g/mL NaCl solution in a single microwell. (**e**) Crystals prepared from saturated NaCl solution (0.36 g/mL) in a single microwell.

We further investigated the influence of NaCl concentration on crystallization. As demonstrated in Figure 7a–c, NaCl crystals were prepared with other concentrations, including 0.27, 0.18, and 0.09 g/mL, in comparison with the saturated concentration (0.36 g/mL). The growth period of the crystals increased significantly for these three concentrations, with 0.09 g/mL exhibiting an almost 20-day growth period. As indicated by a previous report [57], the process of crystallization is influenced by the presence of pre-existing crystals. In the event of crystalline nucleation occurring within the droplet, its subsequent shrinkage will be accelerated relative to that of a droplet devoid of crystals. Saturated solutions have been observed to nucleate at a faster rate when the droplets are undergoing contraction, while the nucleation process in unsaturated solutions is characterized by a substantially longer duration. Consequently, the crystallization growth period of unsaturated solutions is notably extended. The probability of crystals detaching from the droplet and growing outside the droplet increases due to the reduced size of the droplet at the time of nucleation of the low-concentration solution. Many crystals prepared from the 0.09 g/mL solution are less intact, and the probability of multiple crystals or broken crystals occurring in the microwells increases. In comparison with the crystals obtained from the saturated solution, the volume of crystals was marginally diminished at the lower concentration. The discrepancy in the volumes of crystals prepared at the 0.18 g/mL and 0.27 g/mL concentrations is not fully discernible through observation due to the inherent difficulty in assessing the degree of downward growth of crystals. However, the volume of crystals prepared at 0.09 g/mL was notably smaller than that at the saturated concentration, as illustrated in Figure 7d,e.

**Figure 8:**
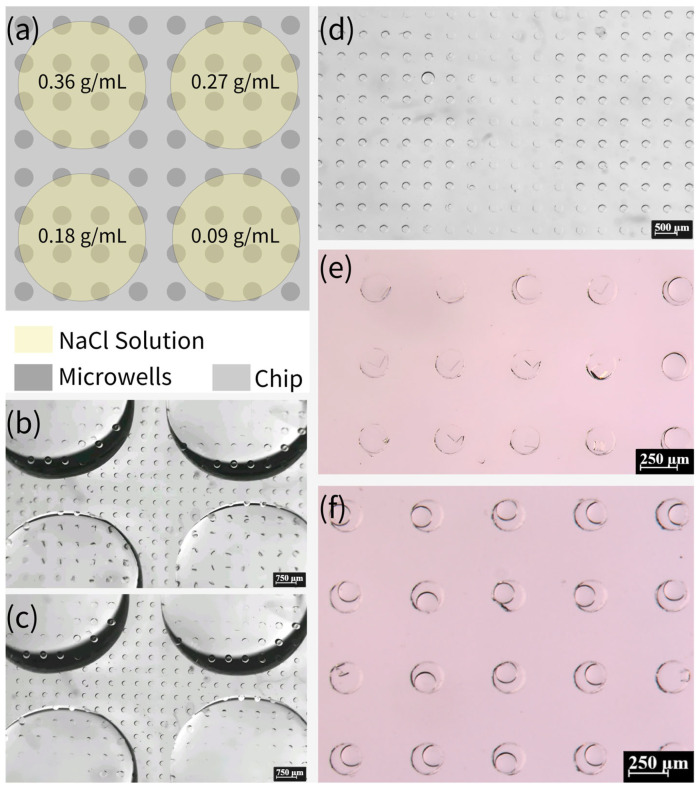
(**a**) Schematic of adding NaCl solutions of different concentrations to the same device. (**b**,**c**) Absorption of NaCl solution into the microwells in the area covered with the solution. (**d**) Clear distinction between the area with and without NaCl solution after covering with mineral oil, where the left and right parts of the picture are the microwells that have absorbed NaCl solution and the middle part is the microwells that have not absorbed NaCl solution. (**e**) Formation of crystals in the area with NaCl solution (0.18 g/mL). (**f**) The area with NaCl solution (0.09 g/mL) has very few microwells with crystals formed, and the droplets in most of the microwells are still continuously shrinking.

Moreover, we investigated the possibility of high-throughput crystallization on this device. Subsequent to the completion of the degassing process, NaCl solutions with varying concentrations (0.09, 0.18, 0.27, and 0.36 g/mL) were added in a dropwise manner to specific locations on the chip. It is imperative to ensure that the solutions of different concentrations do not come into contact with each other, as demonstrated in Figure 8a–c. After five minutes, it is evident that the solutions had been fully absorbed into the microwells. The device was then immediately sealed with mineral oil, as illustrated in Figure 8d. Figure 8e,f presents the results obtained after 15 days. This method of preparing crystals from NaCl solutions of different concentrations on the same device has the potential to facilitate high-throughput crystallization.

### 4.4. Crystallization of Glycine

**Figure 9:**
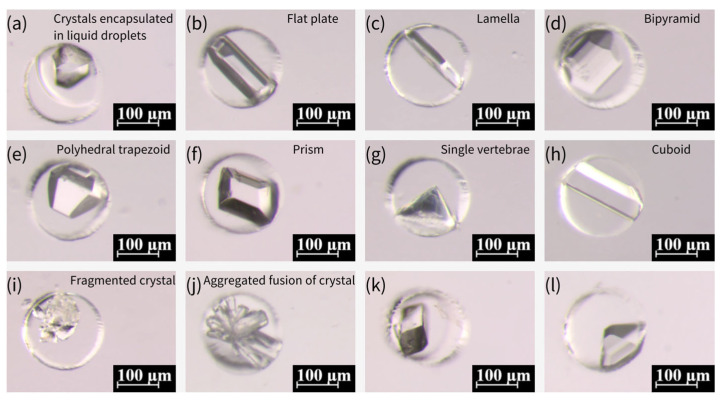
Various morphologies of glycine crystals formed under different temperature conditions. (**a**–**h**) Glycine crystals formed at 50 °C, exhibiting diverse morphological characteristics of α-type and γ-type glycine, including flat plate, lamella, bipyramid, polyhedral trapezoid, prism, single vertebrae, and cuboid forms. (**i**–**l**) Glycine crystals formed at 20 °C, showing fragmented crystal (**i**), aggregated fusion (**j**), and smooth, stable growth of α-type glycine crystals (**k**,**l**).

The same process was used to prepare glycine crystals at 20 °C. The process for generating glycine crystals at 50 °C was slightly different: Firstly, a saturated solution of glycine was prepared in a water bath at 50 °C, given the effect of temperature on the solubility of glycine. Following the degassing of the PDMS microwell array chip, the chip was heated at 50 °C for 5 min. Thereafter, the glycine solution was poured onto the chip, and the chip was placed in a water bath at 50 °C. Following a period of 20 min, the chip was covered with mineral oil to remove the glycine solution from the surface and was continued to be stored in a water bath at 50 °C for 30 min. Subsequently, the chip was taken out from the water bath and placed at 20 °C. Following a period of 6–7 days (for crystallization at 20 °C and 50 °C), it was observed that the glycine crystals were formed inside the microwells and exhibited divergent morphologies, as illustrated in Figure 9.

**Figure 10:**
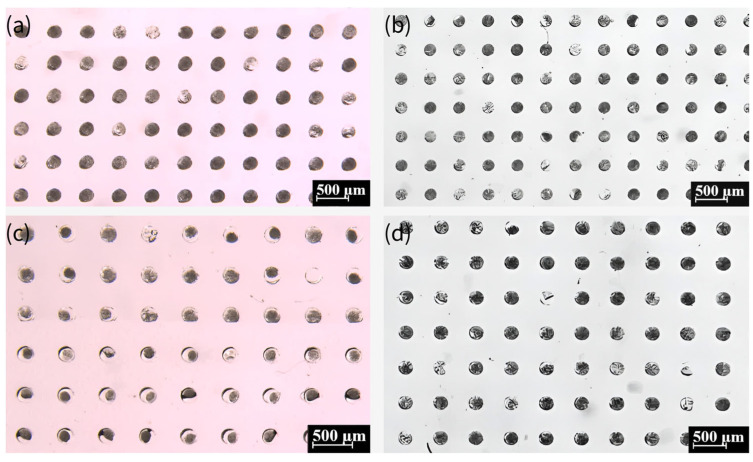
The crystals formed from a saturated glycine solution prepared at 20 °C (**a**), 25 °C (**b**), 35 °C (**c**), and 50 °C (**d**). Black dots show spherical aggregates (SAs).

The crystallization of various crystalline forms of glycine is predominantly influenced by the crystallization position during the growth process [57]. In instances where nuclei are present within the droplet throughout the growth process, the formation of α-type crystals is more probable. Conversely, if some of the nuclei come into contact with the droplet’s boundary during the growth process, the likelihood of generating γ-type crystals is increased. This process exhibits a greater degree of stochasticity. Furthermore, an additional form of spherical aggregates (SAs) was identified for glycine crystals, which were produced by nucleation at multiple locations within the droplet under conditions of high supersaturation. These SAs subsequently coalesced during the process of growth, ultimately resulting in the formation of SAs. According to the extant literature [58], SAs can be generated by subjecting the emulsion to a glass slide of 84 °C. However, alternative sources have also posited that SAs can be produced from saturated glycine solutions prepared at room temperature [57]. An experiment was conducted in which different temperatures were designed and verified to be capable of generating SAs at varying temperatures. The results of this experiment are illustrated in Figure 10a–d, which show that SAs can be generated at different temperatures. It is notable that the crystals prepared at various temperatures could be stored for more than two months and were well-preserved due to the encapsulation of mineral oil.

In comparison with previous microfluidic devices for crystallization, our device is pump-free and relies on degassed PDMS and surface tension for droplet isolation, eliminating pumps or flow control [58,59]. The fabrication of our device is simple, without the need for the assembly of multilayers and 3D printers [60]. In addition, our device demonstrates the possibility of high-throughput crystallization by testing different NaCl concentrations on a single device, beyond the limited droplet control [57].

In short, the microwell array chip demonstrates excellent performance in crystal generation, producing high-quality salt and protein crystals with controlled morphology and stability. By integrating PDMS microwells with passive fluid handling and oil encapsulation, the platform enables robust and polymorphic crystal growth, offering significant potential for drug formulation and biomaterial research. Its scalable design allows for parallel processing of hundreds of crystallization experiments while maintaining precise nucleation control, with minimal occurrences of multiple crystals per microwell.

## 5. Conclusions

This paper sets out a new process for fabricating microfluidic devices, employing polymer OSTE as a replicate mold for conventional microfluidic material PDMS. A coating of Parylene-C is prepared on the surface of polymer OSTE, thus shielding the influence of its surface thiol groups on the curing of PDMS. The PDMS micropillar array and microwell array fabricated by this process are then used in the fabrication of plasma separation chips and the preparation of polymorphic crystals, respectively. The experimental results demonstrate that the plasma separation chips fabricated by this method could effectively separate plasma from whole blood samples with varying HCT in an efficient fashion. Additionally, the microwell array chip, when used as a platform for the preparation of polymorphic crystals, exhibits remarkable stability. The integration of Parylene-C’s chemical stability with OSTE’s mechanical properties offers a reusable and scalable fabrication method. This substantiates the profound potential of this microfluidic device fabrication process in the domains of point-of-care diagnostics and materials synthesis.

## Data Availability

The data are available upon request to guoweijin@stu.edu.cn.

## References

[1] Whitesides G.M. (2006). The origins and the future of microfluidics. Nature.

[2] Sackmann E.K., Fulton A.L., Beebe D.J. (2014). The present and future role of microfluidics in biomedical research. Nature.

[3] Xia Y., Whitesides G.M. (1998). Soft lithography. Angew. Chem. Int. Ed..

[4] Zhang Y., Nguyen N.T. (2017). Magnetic digital microfluidics—A review. Lab Chip.

[5] Ren K., Zhou J., Wu H. (2013). Materials for microfluidic chip fabrication. Acc. Chem. Res..

[6] Juska V.B., Maxwell G., Estrela P., Pemble M.E., O’RIordan A. (2023). Silicon microfabrication technologies for biology integrated advance devices and interfaces. Biosens. Bioelectron..

[7] Stjernström M., Roeraade J. (1998). Method for fabrication of microfluidic systems in glass. J. Micromech. Microeng..

[8] Becker H., Gärtner C. (2007). Polymer microfabrication technologies for microfluidic systems. Anal. Bioanal. Chem..

[9] Van Nguyen T., Nguyen H.M., Nguyen T.X., Tien T.Q., Ta V.D. (2024). Efficient fabrication of high quality SU-8 photoresist based microsphere lasers via emulsion. J. Phys. D Appl. Phys..

[10] Shakeri A., Khan S., Abu Jarad N., Didar T.F. (2022). The fabrication and bonding of thermoplastic microfluidics: A review. Materials.

[11] Nagel J., Weißpflog J., Kroschwald F., Heinrich G. (2015). Tailored chemical surface modifications of different types of thermoplastic materials for microfluidic applications. Macromol. Mater. Eng..

[12] Shakeri A., Abu Jarad N., Khan S., Didar T.F. (2022). Bio-functionalization of microfluidic platforms made of thermoplastic materials: A review. Anal. Chim. Acta.

[13] Musgrove H., Catterton M., Pompano R. (2022). Applied tutorial for the design and fabrication of biomicrofluidic devices by resin 3D printing. Anal. Chim. Acta.

[14] Guttridge C., Shannon A., O’SUllivan A., O’SUllivan K.J., O’SUllivan L.W. (2022). Biocompatible 3D printing resins for medical applications: A review of marketed intended use, biocompatibility certification, and post-processing guidance. Ann. 3D Print. Med..

[15] de Jesus Vieira J.P., da Silva S.S., de Fátima Souza I., Pedras M.B., de Avelar Freitas B.A., Torres L.A.G. (2025). Study of the biocompatibility of polymeric resins processed by 3D printing for applications in manufacturing of devices for short term 3D cultures. Microfluid. Nanofluidics.

[16] Mehta V., Rath S.N. (2021). 3D printed microfluidic devices: A review focused on four fundamental manufacturing approaches and implications on the field of healthcare. Bio-Des. Manuf..

[17] Ahmadianyazdi A., Miller I.J., Folch A. (2023). Tunable resins with PDMS-like elastic modulus for stereolithographic 3D-printing of multimaterial microfluidic actuators. Lab Chip.

[18] Paguirigan A., Beebe D.J. (2006). Gelatin based microfluidic devices for cell culture. Lab Chip.

[19] Sasaki S., Suzuki T., Morikawa K., Matsusaki M., Sato K. (2022). Fabrication of a Gelatin-Based Microdevice for Vascular Cell Culture. Micromachines.

[20] Rosellini E., Cascone M.G. (2023). Microfluidic Fabrication of Natural Polymer-Based Scaffolds for Tissue Engineering Applications: A Review. Biomimetics.

[21] Li H.F., Lin J.M., Su R.G., Cai Z.W., Uchiyama K. (2005). A polymeric master replication technology for mass fabrication of poly (dimethylsiloxane) microfluidic devices. Electrophoresis.

[22] Senn T., Esquivel J.P., Lörgen M., Sabaté N., Löchel B. (2010). Replica molding for multilevel micro-/nanostructure replication. J. Micromech. Microeng..

[23] Borysiak M.D., Bielawski K.S., Sniadecki N.J., Jenkel C.F., Vogt B.D., Posner J.D. (2013). Simple replica micromolding of biocompatible styrenic elastomers. Lab Chip.

[24] Chandra D., Taylor J.A., Yang S. (2008). Replica molding of high-aspect-ratio (sub-)micron hydrogel pillar arrays and their stability in air and solvents. Soft Matter.

[25] Li Y., Ng H.W., Gates B.D., Menon C. (2014). Material versatility using replica molding for large-scale fabrication of high aspect-ratio, high density arrays of nano-pillars. Nanotechnology.

[26] Scott S.M., Ali Z. (2021). Fabrication methods for microfluidic devices: An overview. Micromachines.

[27] Carlborg C.F., Haraldsson T., Öberg K., Malkoch M., van der Wijngaart W. (2011). Beyond PDMS: Off-stoichiometry thiol–ene (OSTE) based soft lithography for rapid prototyping of microfluidic devices. Lab Chip.

[28] Sandström N., Shafagh R.Z., Vastesson A., Carlborg C.F., van der Wijngaart W., Haraldsson T. (2015). Reaction injection molding and direct covalent bonding of OSTE+ polymer microfluidic devices. J. Micromech. Microeng..

[29] Zhang M., Li H., Xiao Z., Feng Z., Yu S., Chen Z., Zhang H., Guo W. Fabrication of Concave Microwells and Microchannels by Off-stoichiometry Thiol-ene (OSTE) Backside Lithography. Proceedings of the 2023 IEEE 18th International Conference on Nano/Micro Engineered and Molecular Systems (NEMS).

[30] Zandi Shafagh R., Vastesson A., Guo W., Van Der Wijngaart W., Haraldsson T. (2018). E-beam nanostructuring and direct click biofunctionalization of thiol–ene resist. ACS Nano.

[31] Guo W., Hansson J., Gustafsson L., van der Wijngaart W. “Bend-and-Bond” Polymer Microfluidic Origami. Proceedings of the 2021 IEEE 34th International Conference on Micro Electro Mechanical Systems (MEMS).

[32] Guo W., Gustafsson L., Jansson R., Hedhammar M., van der Wijngaart W. Formation of a thin-walled spider silk tube on a micromachined scaffold. Proceedings of the 2018 IEEE Micro Electro Mechanical Systems (MEMS).

[33] Li H., Zhang M., Liu Y., Yu S., Li X., Chen Z., Feng Z., Zhou J., He Q., Chen X. (2024). Off-Stoichiometry Thiol-Ene (OSTE) Micro Mushroom Forest: A Superhydrophobic Substrate. Micromachines.

[34] Gorham W.F. (1966). A new, general synthetic method for the preparation of linear poly-p-xylylenes. J. Polym. Sci. Part. A 1 Polym. Chem..

[35] Golda-Cepa M., Engvall K., Hakkarainen M., Kotarba A. (2020). Recent progress on parylene C polymer for biomedical applications: A review. Prog. Org. Coat..

[36] Ortigoza-Diaz J., Scholten K., Larson C., Cobo A., Hudson T., Yoo J., Baldwin A., Hirschberg A.W., Meng E. (2018). Techniques and Considerations in the Microfabrication of Parylene C Microelectromechanical Systems. Micromachines.

[37] Rodger D.C., Fong A.J., Li W., Ameri H., Ahuja A.K., Gutierrez C., Lavrov I., Zhong H., Menon P.R., Meng E. (2008). Flexible parylene-based multielectrode array technology for high-density neural stimulation and recording. Sens. Actuators B Chem..

[38] Xie X., Rieth L., Williams L., Negi S., Bhandari R., Caldwell R., Sharma R., Tathireddy P., Solzbacher F. (2014). Long-term reliability of Al_2_O_3_ and Parylene C bilayer encapsulated Utah electrode array based neural interfaces for chronic implantation. J. Neural Eng..

[39] Zhang Y.N., Sun P., Zhou C., Jia M. Studying of parylene film on microwave loading performance in aerospace products. Proceedings of the 2022 23rd International Conference on Electronic Packaging Technology (ICEPT).

[40] Blahová L., Horák J., Přikryl R., Pekárek J., Tkacz J., Menčík P., Krčma F. (2019). A novel technology for the corrosion protection of iron archaeological artefacts using parylene base removable bilayer. J. Cult. Heritage.

[41] Fang X., Sun C., Dai P., Xian Z., Su W., Zheng C., Xing D., Xu X., You H. (2024). Capillary Force-Driven Quantitative Plasma Separation Method for Application of Whole Blood Detection Microfluidic Chip. Micromachines.

[42] Bastin R.J., Bowker M.J., Slater B.J. (2000). Salt Selection and Optimisation Procedures for Pharmaceutical New Chemical Entities. Org. Process. Res. Dev..

[43] Quilaqueo M., Aguilera J.M. (2016). Crystallization of NaCl by fast evaporation of water in droplets of NaCl solutions. Food Res. Int..

[44] Rabesiaka M., Sghaier M., Fraisse B., Porte C., Havet J.-L., Dichi E. (2010). Preparation of glycine polymorphs crystallized in water and physicochemical characterizations. J. Cryst. Growth.

[45] Yu L., Ng K. (2002). Glycine Crystallization during Spray Drying: The pH Effect on Salt and Polymorphic Forms. J. Pharm. Sci..

[46] Xu L., Lee H., Jetta D., Oh K.W. (2015). Vacuum-driven power-free microfluidics utilizing the gas solubility or permeability of polydimethylsiloxane (PDMS). Lab Chip.

[47] Heyries K.A., Hansen C.L. (2011). Parylene C coating for high-performance replica molding. Lab Chip.

[48] Chen Y., Pei W., Tang R., Chen S., Chen H. (2013). Conformal coating of parylene for surface anti-adhesion in polydimethylsiloxane (PDMS) double casting technique. Sensors Actuators A Phys..

[49] Martinez-Duarte R., Madou M. (2011). SU-8 photolithography and its impact on microfluidics. Microfluidics and Nanofluidics Handbook.

[50] Das A., Sinha A., Rao V., Jonnalagadda K. (2017). Fracture in Microscale SU-8 Polymer Thin Films. Exp. Mech..

[51] Hillmering M., Pardon G., Vastesson A., Supekar O., Carlborg C.F., Brandner B.D., van der Wijngaart W., Haraldsson T. (2016). Off-stoichiometry improves the photostructuring of thiol–enes through diffusion-induced monomer depletion. Microsyst. Nanoeng..

[52] Ejserholm F., Stegmayr J., Bauer P., Johansson F., Wallman L., Bengtsson M., Oredsson S. (2015). Biocompatibility of a polymer based on Off-Stoichiometry Thiol-Enes + Epoxy (OSTE+) for neural implants. Biomater. Res..

[53] Chen Z., Li H., Zhang M., Li X., Zhang Y., Zhu G., Feng Z., Xiao Z., Zhang H., Cui X. (2024). Cotton threads encapsulated by thermal contraction tube for point-of-care diagnostics. Microchem. J..

[54] Zhang H., Anoop K., Huang C., Sadr R., Gupte R., Dai J., Han A. (2022). A circular gradient-width crossflow microfluidic platform for high-efficiency blood plasma separation. Sens. Actuators B Chem..

[55] Xiao Z., Sun L., Yang Y., Feng Z., Dai S., Yang H., Zhang X., Sheu C.-L., Guo W. (2021). High-Performance Passive Plasma Separation on OSTE Pillar Forest. Biosensors.

[56] Guo W., Hansson J., van der Wijngaart W. (2020). Synthetic Paper Separates Plasma from Whole Blood with Low Protein Loss. Anal. Chem..

[57] Yang S.M., Zhang D., Chen W., Chen S.C. (2015). A flow-free droplet-based device for high throughput polymorphic crystallization. Lab Chip.

[58] Toldy A.I., Badruddoza A.Z.M., Zheng L., Hatton T.A., Gunawan R., Rajagopalan R., Khan S.A. (2012). Spherical Crystallization of Glycine from Monodisperse Microfluidic Emulsions. Cryst. Growth Des..

[59] Tona R.M., McDonald T.A., Akhavein N., Larkin J.D., Lai D. (2019). Microfluidic droplet liquid reactors for active pharmaceutical ingredient crystallization by diffusion controlled solvent extraction. Lab Chip.

[60] Liang Y.R., Zhu L.N., Gao J., Zhao H.X., Zhu Y., Ye S., Fang Q. (2017). 3D-printed high-density droplet array chip for miniaturized protein crystallization screening under vapor diffusion mode. ACS Appl. Mater. Interfaces.

